# Crystal structure of 5-(4-*tert*-but­oxy­phen­yl)-3-(4-*n*-octyloxyphen­yl)-4,5-di­hydro­isoxazole

**DOI:** 10.1107/S2056989019007412

**Published:** 2019-05-24

**Authors:** Eric S. Sales, Adailton J. Bortoluzzi, Aloir A. Merlo

**Affiliations:** a Instituto de Química, Universidade Federal do Rio Grande do Sul - UFRGS, Av. Bento Gonçalves, 9500, 91501 - 970 - Porto Alegre - RS, Brazil; b Depto. de Química - Campus Trindade, Universidade Federal de Santa Catarina - UFSC, 88040-900 - Florianópolis, Santa Catarina, Brazil

**Keywords:** isoxazolines, liquid crystals, [3 + 2] cyclo­addition, single crystals, crystal structure

## Abstract

The mol­ecule of the title compound, C_27_H_37_NO_3_, was prepared by [3 + 2] 1,3-dipolar cyclo­addition of 4-*n*-octyl­phenyl­nitrile oxide and 4-*tert*-but­oxy­styrene, the latter compound being a very useful inter­mediate to the synthesis of liquid-crystalline materials. In the mol­ecule, the benzene rings of the *n*-octyloxyphenyl and *tert*-but­oxy­phenyl groups form dihedral angles of 2.83 (7) and 85.49 (3)°, respectively, with the mean plane of the isoxazoline ring. In the crystal, mol­ecules are linked by weak C—H⋯O hydrogen inter­actions into chains running parallel to the *b* axis.

## Abstract 2

Δ^2^-Isoxazolines constitute an important class of five-membered heterocycles which have significant synthetic and biological applications. Herein is presented a concise route to the synthesis of liquid crystals based on isoxazolines and structural characterization of 5-[4-(*tert*-but­oxy)phen­yl]-3-[4-(n-oct­yloxy)phen­yl]-4,5-di-hydro­isoxazole.

## Chemical context   

Nitro­gen- and oxygen-containing heterocycles known as Δ^2^-isoxazolines constitute an important class of five-membered heterocycles which have significant synthetic and biological applications (Pirrung *et al.*, 2002 ▸; Choe *et al.*, 2016 ▸; Huang *et al.*, 2017 ▸; Stosic-Grujicic *et al.*, 2007 ▸). Isoxazolines display diverse biological and pharmacological properties. This unique class of pharmacophores occurs naturally in many therapeutic agents. The chlorinated isoxazoline anti­tumor anti­biotics U-42,126 and U-43,795 isolated from *Streptomyces sviceus*, exhibit significant activity against L 1210 lymphoid leukaemia in mice (Martin *et al.*, 1975 ▸; Hanka *et al.*, 1975 ▸). Inspired by this class of natural anti­biotics, a new library of natural products probes have been designed, synthesized and tested for bacterial proteome analysis (Orth *et al.*, 2010 ▸). Nitro­furan­ylisoxazolines with increased proteolytic stability have been investigated, leading to the discovery of several compounds with potent *in vitro* anti-tuberculosis activity (Tangallapally *et al.*, 2007 ▸). Trihalomethyl-pyrimidine sugar-modified nucleosides containing the isoxazoline ring were synthesized and their *in vitro* anti­proliferactive activity evaluated against human cancer cell lines and one of them was three times more selective than MXT standard anti­cancer drugs (Lobo *et al.*, 2015 ▸). Isoxazolines have proven be an excellent GABA receptors, as demonstrated by Ozoe *et al.* (2010 ▸) who reported isoxazoline A1443 to exhibit anti­parasitic activity against cat fleas and dog ticks comparable to that of the commercial ectoparasiticide fipronil. From a synthetic point of view, Δ^2^-isoxazolines constitute an important way to synthesize many natural products with diverse and intricate mol­ecular connectivity. Bafilomycin A1 and erythromycin A, reported by the Carreira group, are examples of the versatility of isoxazoline in the total synthesis of natural products (Kleinbeck & Carreira 2009 ▸; Muri & Carreira 2009 ▸).

Previously we have demonstrated that [3 + 2] 1,3-dipolar cyclo­addition of aryl­nitrile oxide to alkene is a excellent route to access different 3,5-disubstituted isoxazolines (Tavares *et al.*, 2010 ▸, 2016 ▸; Fritsch & Merlo, 2016 ▸; Lopes *et al.*, 2018 ▸). Using this methodology, a collection of isoxazolines can be constructed with specific applications ranging from biological compounds through use as inter­mediates in organic synthesis to liquid-crystal materials (El-Khatatneh *et al.*, 2017 ▸; Fader & Carreira, 2004 ▸; Bezborodov *et al.*, 2004 ▸). With this purpose in mind, we have established a concise route to the synthesis of liquid crystals based on isoxazolines and their full characterization. The [3 + 2] 1,3-dipolar cyclo­addition requires two partners, one being nitrile oxide (1,3-dipole) obtained from oxime correspondent and other is an alkene (Huisgen, 1976 ▸). Thus, considering the liquid crystals thematic, we focused our attention on the preparation of distorted rod-shaped mol­ecules based on isoxazolines using 4-*t*-but­oxy­phenyl styrene as the dipholarophile and 4-*n*-alk­oxy­phenyl nitrile oxide as the 1,3-dipole. The title compound was synthesized in three steps starting from 4-hy­droxy­benzaldehyde by alkyl­ation reaction (85% yield), oximation reaction (89% yield) and [3 + 2] 1,3-dipolar cyclo­addition (51% yield).
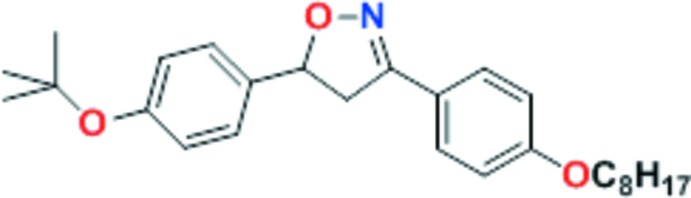


## Structural commentary   

**Figure 1:**
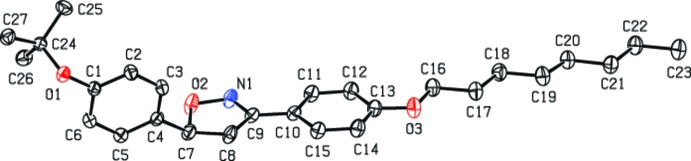
*ORTEP* plot of the title compound showing displacement ellipsoids drawn at the 40% probability level. Hydrogen atoms are omitted for clarity.

In the mol­ecule of the title compound (Fig. 1 ▸), the isoxazoline ring adopts a twist conformation, with puckering parameters *q*_2_ = 0.1522 (11) Å and Φ_2_ = 149.6 (4)°. The mean plane through the isoxazoline ring [maximum deviation 0.1113 (12) Å for atom C7] is approximately coplanar with the C10–C15 aromatic ring of the *n*-octyloxyphenyl group [dihedral angle = 2.83 (7)°], whereas it is almost perpendicular to the C1–C6 benzene ring of the *t*-but­oxy­phenyl group [dihedral angle = 85.49 (3)°]. The C16–C23 aliphatic chain shows a regular extended conformation.

## Supra­molecular features   

**Figure 2:**
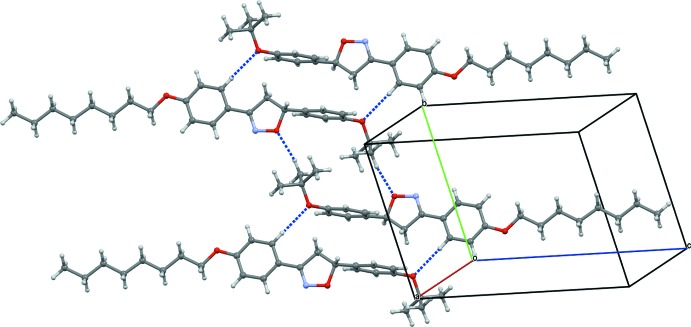
Hydrogen-bonding inter­actions (dashed lines) in the title compound.

In the crystal, mol­ecules of Δ^2^-isoxazolines are accommodated in sheets parallel to (010). In each sheet, centrosymmetrically related mol­ecules are connected by a pair of weak non-classical C—H⋯O hydrogen bonds (Table 1 ▸), forming dimeric units (Fig. 2 ▸), which are further linked into chains parallel to the *b* axis by weak C—H⋯O hydrogen bonds involving the oxygen atoms of the *t*-but­oxy group as acceptors. No C—H⋯π contacts or π–π inter­actions involving the benzene rings of the 3,5-di­aryl­isoxazoline system are observed.

## Database survey   

A search of the 3,5-di­aryl­isoxazoline moiety revealed 22 entries in the Cambridge Structural Database (Version 2.0.1, update of February 2019; Groom *et al.*, 2016 ▸). However, when the search was restricted to *para*-diether-3,5-di­aryl­isoxazoline, just one entry was retrieved. The match AWUYUN is associated with the work published by Samshuddin *et al.* (2011 ▸), which describes the crystal structure of 3,5-*bis*(4-meth­oxy­phen­yl)-4,5-di­hydro­isoxazole. In both cases, the five-membered isoxazoline ring is coplanar with the phenyl ring bonded to the nitro­gen side, whereas the phenyl ring on the oxygen side is very twisted, with dihedral angles between the mean planes of the phenyl rings close to orthogonal.

## Synthesis and crystallization   

4-(*n*-Oct­yloxy)benzaldehyde and 4-(*n-*oct­yloxy)benzaldehyde oxime were prepared according to the procedures reported by Passo *et al.* (2008 ▸) and Tavares *et al.* (2009 ▸). The general procedure for the preparation of 5-[4-(*tert*-but­oxy)phen­yl]-3-[4-(oct­yloxy)phen­yl]-4,5-di­hydro­isoxazole is described as follows: To a solution of 4-*n*-octyloxybenzaldehyde oxime (5 mmol, 1,246 g) and *N*-chloro­succinimide (5.35 mmol, 0.72 g) in THF (40 mL) was added 1 drop of concentrated HCl. The final solution was stirred by additional 30 min and cooled to 273 K. Then 4-*tert*-but­oxy­stirene (5 mmol, 0.9 mL) in tri­ethyl­amine (15 mmol, 2.1 mL) was added dropwise, followed by stirring for one h at room temperature. The final solution was filtered and THF was removed by evaporation. The crude product was dissolved in CH_2_Cl_2_ (2 ×100mL) and washed with 1 *M* HCl (3 × 50 mL), saturated NaHCO_3_ (1 × 50 mL) and brine (1 × 50 mL). The organic solution was dried over Na_2_SO_4_, the solvent was removed by evaporation and the yellow solid was recrystallized in ethanol. Single crystals of the title compound were collected on slow evaporation of the solvent. Data collected for 5-[4-(*tert*-but­oxy)phen­yl)-3-[4-(*n*-oct­yloxy)phen­yl]-4,5-di­hydro-isoxazole: white solid; yield: 51%; m.p. 335–337 K; ^1^H NMR (300 MHz, CDCl_3_), δ (ppm): 7.65–7.58 (*m*, 2H), 7.32–7.26 (*m*, 2H), 7.02–6.95 (*m*, 2H), 6.95–6.88 (*m*, 2H), 5.66 (*dd*, *J*_cis_ = 10.8 Hz, *J*_trans_ = 8.5 Hz, 1H), 3.98 (*t*, *J* = 6.6 Hz, 2H), 3.71 (*dd*, *J*_gem_ = 16.6 Hz, *J*_cis_ = 10.8 Hz, 1H), 3.32 (*dd*, *J*_gem_ = 16.6 Hz, *J*_trans_ = 8.5 Hz, 1H), 1.84–1.72 (*m*, 2H), 1.53–1.19 (*m*, 19H), 0.93–0.83 (*m*, 3H); ^13^C NMR (75 MHz, CDCl_3_), δ (ppm): 160.8, 156.0, 155.5, 135.8, 128.4, 126.8, 124.5, 121.9, 114.8, 82.3, 78.8, 77.4, 68.3, 43.4, 31.9, 29.5, 29.4, 29.3, 29.0, 26.1, 22.8, 14.3 (1 signal is missing).

## Refinement   

Selected crystal data, data collection and structure refinement details are summarized in Table 2 ▸. All hydrogen atoms were positioned geometrically using a riding atom approximation, with C—H = 0.95–1.00 Å, and with *U*_iso_(H) = 1.2*U*_eq_(C) or 1.5*U*_eq_(C) for methyl H atoms. A rotating model was used for the methyl groups.

## Supplementary Material

Crystal structure: contains datablock(s) I, New_Global_Publ_Block. DOI: 10.1107/S2056989019007412/rz5256sup1.cif

Structure factors: contains datablock(s) I. DOI: 10.1107/S2056989019007412/rz5256Isup2.hkl

Supporting information file. DOI: 10.1107/S2056989019007412/rz5256Isup3.mol

Supporting information file. DOI: 10.1107/S2056989019007412/rz5256Isup4.cml

CCDC reference: 1917652

Additional supporting information:  crystallographic information; 3D view; checkCIF report

## Figures and Tables

**Table 1:** Hydrogen-bond geometry (Å, °)

*D*—H⋯*A*	*D*—H	H⋯*A*	*D*⋯*A*	*D*—H⋯*A*
C26—H26*B*⋯O2^i^	0.98	2.56	3.4652 (14)	154
C15—H15⋯O1^ii^	0.95	2.61	3.5542 (12)	173

Symmetry codes: (i) 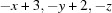; (ii) 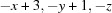.

**Table 2:** Experimental details

Crystal data	
Chemical formula	C_27_H_37_NO_3_
*M*_r_	423.57
Crystal system, space group	Triclinic, *P* 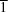
Temperature (K)	173
*a*, *b*, *c* (Å)	5.8493 (1), 10.7773 (3), 19.3201 (6)
α, β, γ (°)	92.325 (1), 91.806 (1), 94.145 (1)
*V* (Å^3^)	1213.02 (5)
*Z*	2
Radiation type	Mo *K*α
μ (mm^−1^)	0.07
Crystal size (mm)	0.50 × 0.20 × 0.12

Data collection	
Diffractometer	Bruker APEXII DUO
Absorption correction	Multi-scan (*SADABS*; Bruker, 2012 ▸)
*T*_min_, *T*_max_	0.711, 0.747
No. of measured, independent and observed [*I* > 2σ(*I*)] reflections	10857, 7607, 6342
*R*_int_	0.009
(sin θ/λ)_max_ (Å^−1^)	0.725

Refinement	
*R*[*F* ^2^ > 2σ(*F* ^2^)], *wR*(*F* ^2^), *S*	0.049, 0.140, 1.03
No. of reflections	7607
No. of parameters	284
H-atom treatment	H-atom parameters constrained
Δρ_max_, Δρ_min_ (e Å^−3^)	0.40, −0.19

Computer programs: *APEX2* and *SAINT* (Bruker, 2012 ▸), *SHELXTL* (Sheldrick, 2008 ▸), *SHELXL2018* (Sheldrick, 2015 ▸), *PLATON* (Spek, 2009 ▸), *Mercury* (Macrae *et al.*, 2008 ▸) and *publCIF* (Westrip, 2010 ▸).

## supplementary crystallographic information

### Crystal data

**Table:**

C_27_H_37_NO_3_	*Z* = 2
*M**_r_* = 423.57	*F*(000) = 460
Triclinic, *P*1	*D*_x_ = 1.160 Mg m^−^^3^
*a* = 5.8493 (1) Å	Mo *K*α radiation, λ = 0.71073 Å
*b* = 10.7773 (3) Å	Cell parameters from 7320 reflections
*c* = 19.3201 (6) Å	θ = 2.8–34.2°
α = 92.325 (1)°	µ = 0.07 mm^−^^1^
β = 91.806 (1)°	*T* = 173 K
γ = 94.145 (1)°	Block, colourless
*V* = 1213.02 (5) Å^3^	0.50 × 0.20 × 0.12 mm

### Data collection

**Table:**

Bruker APEXII DUO diffractometer	7607 independent reflections
Radiation source: fine-focus sealed tube	6342 reflections with *I* > 2σ(*I*)
Graphite monochromator	*R*_int_ = 0.009
φ and ω scans	θ_max_ = 31.0°, θ_min_ = 2.9°
Absorption correction: multi-scan (SADABS; Bruker, 2012)	*h* = −7→8
*T*_min_ = 0.711, *T*_max_ = 0.747	*k* = −15→15
10857 measured reflections	*l* = −27→27

### Refinement

**Table:**

Refinement on *F*^2^	Primary atom site location: structure-invariant direct methods
Least-squares matrix: full	Secondary atom site location: difference Fourier map
*R*[*F*^2^ > 2σ(*F*^2^)] = 0.049	Hydrogen site location: inferred from neighbouring sites
*wR*(*F*^2^) = 0.140	H-atom parameters constrained
*S* = 1.03	*w* = 1/[σ^2^(*F*_o_^2^) + (0.0667*P*)^2^ + 0.4021*P*] where *P* = (*F*_o_^2^ + 2*F*_c_^2^)/3
7607 reflections	(Δ/σ)_max_ = 0.001
284 parameters	Δρ_max_ = 0.40 e Å^−^^3^
0 restraints	Δρ_min_ = −0.19 e Å^−^^3^

### Special details

**Table:**

Geometry. All esds (except the esd in the dihedral angle between two l.s. planes) are estimated using the full covariance matrix. The cell esds are taken into account individually in the estimation of esds in distances, angles and torsion angles; correlations between esds in cell parameters are only used when they are defined by crystal symmetry. An approximate (isotropic) treatment of cell esds is used for estimating esds involving l.s. planes.

### Fractional atomic coordinates and isotropic or equivalent isotropic displacement parameters (Å^2^)

**Table:**

	*x*	*y*	*z*	*U*_iso_*/*U*_eq_	
C1	1.64769 (16)	0.74894 (8)	−0.05545 (5)	0.02252 (17)	
C2	1.43190 (17)	0.69199 (10)	−0.04304 (5)	0.02649 (19)	
H2	1.318117	0.680926	−0.079384	0.032*	
C3	1.38402 (17)	0.65151 (10)	0.02272 (5)	0.0276 (2)	
H3	1.236687	0.613066	0.031010	0.033*	
C4	1.54893 (17)	0.66646 (9)	0.07669 (5)	0.02462 (18)	
C5	1.76598 (18)	0.71882 (10)	0.06288 (6)	0.0287 (2)	
H5	1.881608	0.727313	0.098755	0.034*	
C6	1.81591 (17)	0.75896 (10)	−0.00292 (5)	0.0275 (2)	
H6	1.965700	0.793326	−0.011855	0.033*	
C7	1.4927 (2)	0.63786 (10)	0.14996 (6)	0.0298 (2)	
H7	1.639120	0.635864	0.177901	0.036*	
C8	1.3386 (2)	0.52173 (10)	0.16367 (6)	0.0331 (2)	
H8A	1.257332	0.486755	0.120734	0.040*	
H8B	1.426563	0.456841	0.184659	0.040*	
C9	1.17552 (18)	0.57422 (9)	0.21388 (5)	0.02437 (18)	
C10	1.00340 (17)	0.50153 (9)	0.25159 (5)	0.02354 (18)	
C11	0.85608 (19)	0.56078 (9)	0.29567 (5)	0.0283 (2)	
H11	0.870372	0.649014	0.301232	0.034*	
C12	0.6903 (2)	0.49430 (10)	0.33136 (6)	0.0305 (2)	
H12	0.590952	0.536589	0.360612	0.037*	
C13	0.66989 (19)	0.36438 (10)	0.32407 (5)	0.0277 (2)	
C14	0.8169 (2)	0.30396 (10)	0.28077 (6)	0.0305 (2)	
H14	0.804578	0.215616	0.276084	0.037*	
C15	0.98029 (19)	0.37134 (9)	0.24460 (5)	0.0282 (2)	
H15	1.077691	0.328959	0.214763	0.034*	
C16	0.3577 (2)	0.34639 (11)	0.40164 (6)	0.0317 (2)	
H16A	0.264382	0.402808	0.375405	0.038*	
H16B	0.442735	0.395495	0.439870	0.038*	
C17	0.2055 (2)	0.24281 (11)	0.43018 (6)	0.0335 (2)	
H17A	0.302437	0.184383	0.453525	0.040*	
H17B	0.119058	0.196173	0.391341	0.040*	
C18	0.0366 (2)	0.29097 (11)	0.48151 (6)	0.0330 (2)	
H18A	−0.060442	0.349489	0.458264	0.040*	
H18B	0.122729	0.337310	0.520501	0.040*	
C19	−0.1167 (2)	0.18553 (11)	0.50984 (6)	0.0349 (2)	
H19A	−0.208792	0.142655	0.470892	0.042*	
H19B	−0.018349	0.124293	0.530006	0.042*	
C20	−0.2783 (2)	0.22801 (10)	0.56467 (6)	0.0318 (2)	
H20A	−0.379620	0.287585	0.544232	0.038*	
H20B	−0.186764	0.272574	0.603192	0.038*	
C21	−0.4267 (2)	0.12127 (10)	0.59381 (6)	0.0327 (2)	
H21A	−0.508740	0.072300	0.554987	0.039*	
H21B	−0.326314	0.065477	0.617931	0.039*	
C22	−0.6008 (2)	0.16621 (11)	0.64401 (6)	0.0331 (2)	
H22A	−0.699866	0.222595	0.619849	0.040*	
H22B	−0.518114	0.214970	0.682803	0.040*	
C23	−0.7519 (3)	0.06135 (14)	0.67351 (7)	0.0447 (3)	
H23A	−0.837883	0.013891	0.635584	0.067*	
H23B	−0.859495	0.096688	0.705400	0.067*	
H23C	−0.655669	0.006054	0.698516	0.067*	
C24	1.65820 (16)	0.91821 (9)	−0.13541 (5)	0.02269 (17)	
C25	1.40381 (18)	0.93736 (11)	−0.13308 (6)	0.0317 (2)	
H25A	1.317724	0.874246	−0.163469	0.047*	
H25B	1.376322	1.020479	−0.148808	0.047*	
H25C	1.353249	0.929723	−0.085465	0.047*	
C26	1.7923 (2)	1.00842 (10)	−0.08406 (6)	0.0312 (2)	
H26A	1.738292	0.994948	−0.037262	0.047*	
H26B	1.769305	1.094089	−0.096335	0.047*	
H26C	1.955819	0.994439	−0.085452	0.047*	
C27	1.7451 (2)	0.93263 (11)	−0.20797 (5)	0.0308 (2)	
H27A	1.909480	0.920386	−0.207896	0.046*	
H27B	1.719501	1.016327	−0.223155	0.046*	
H27C	1.662418	0.870491	−0.239749	0.046*	
N1	1.19161 (18)	0.69382 (8)	0.21931 (5)	0.0310 (2)	
O1	1.69856 (13)	0.78825 (6)	−0.12071 (4)	0.02499 (15)	
O2	1.36545 (16)	0.74135 (7)	0.17716 (4)	0.03545 (19)	
O3	0.51450 (15)	0.28925 (8)	0.35685 (4)	0.03590 (19)	

### Atomic displacement parameters (Å^2^)

**Table:**

	*U*^11^	*U*^22^	*U*^33^	*U*^12^	*U*^13^	*U*^23^
C1	0.0238 (4)	0.0203 (4)	0.0240 (4)	0.0033 (3)	0.0063 (3)	0.0010 (3)
C2	0.0241 (4)	0.0280 (4)	0.0268 (4)	−0.0020 (3)	0.0016 (3)	0.0013 (3)
C3	0.0227 (4)	0.0290 (5)	0.0312 (5)	−0.0010 (3)	0.0053 (4)	0.0046 (4)
C4	0.0273 (4)	0.0212 (4)	0.0263 (4)	0.0042 (3)	0.0056 (3)	0.0051 (3)
C5	0.0256 (4)	0.0315 (5)	0.0291 (5)	0.0007 (4)	0.0000 (4)	0.0070 (4)
C6	0.0211 (4)	0.0300 (5)	0.0316 (5)	0.0004 (3)	0.0031 (3)	0.0065 (4)
C7	0.0351 (5)	0.0285 (5)	0.0271 (5)	0.0050 (4)	0.0069 (4)	0.0079 (4)
C8	0.0458 (6)	0.0206 (4)	0.0353 (5)	0.0092 (4)	0.0192 (5)	0.0071 (4)
C9	0.0314 (5)	0.0211 (4)	0.0214 (4)	0.0048 (3)	0.0044 (3)	0.0024 (3)
C10	0.0294 (4)	0.0211 (4)	0.0205 (4)	0.0035 (3)	0.0030 (3)	0.0011 (3)
C11	0.0361 (5)	0.0224 (4)	0.0268 (4)	0.0034 (4)	0.0077 (4)	−0.0008 (3)
C12	0.0367 (5)	0.0267 (5)	0.0285 (5)	0.0029 (4)	0.0104 (4)	−0.0013 (4)
C13	0.0317 (5)	0.0271 (5)	0.0238 (4)	−0.0011 (4)	0.0044 (4)	0.0004 (3)
C14	0.0388 (6)	0.0214 (4)	0.0312 (5)	0.0000 (4)	0.0074 (4)	−0.0013 (4)
C15	0.0357 (5)	0.0223 (4)	0.0272 (4)	0.0034 (4)	0.0080 (4)	−0.0005 (3)
C16	0.0332 (5)	0.0341 (5)	0.0278 (5)	−0.0007 (4)	0.0072 (4)	0.0018 (4)
C17	0.0329 (5)	0.0352 (5)	0.0320 (5)	−0.0036 (4)	0.0067 (4)	0.0037 (4)
C18	0.0327 (5)	0.0369 (5)	0.0295 (5)	−0.0010 (4)	0.0055 (4)	0.0045 (4)
C19	0.0346 (6)	0.0351 (5)	0.0346 (5)	−0.0028 (4)	0.0097 (4)	−0.0002 (4)
C20	0.0336 (5)	0.0309 (5)	0.0306 (5)	−0.0021 (4)	0.0068 (4)	0.0000 (4)
C21	0.0359 (5)	0.0275 (5)	0.0352 (5)	0.0017 (4)	0.0098 (4)	0.0021 (4)
C22	0.0376 (6)	0.0318 (5)	0.0295 (5)	−0.0019 (4)	0.0084 (4)	−0.0008 (4)
C23	0.0443 (7)	0.0479 (7)	0.0425 (7)	−0.0032 (6)	0.0112 (5)	0.0131 (6)
C24	0.0229 (4)	0.0216 (4)	0.0240 (4)	0.0036 (3)	0.0037 (3)	0.0014 (3)
C25	0.0233 (4)	0.0380 (5)	0.0347 (5)	0.0080 (4)	0.0034 (4)	0.0019 (4)
C26	0.0344 (5)	0.0241 (4)	0.0345 (5)	0.0013 (4)	−0.0036 (4)	−0.0006 (4)
C27	0.0341 (5)	0.0327 (5)	0.0272 (5)	0.0065 (4)	0.0090 (4)	0.0060 (4)
N1	0.0436 (5)	0.0233 (4)	0.0265 (4)	0.0019 (3)	0.0114 (4)	0.0008 (3)
O1	0.0318 (4)	0.0211 (3)	0.0230 (3)	0.0046 (3)	0.0093 (3)	0.0011 (2)
O2	0.0524 (5)	0.0219 (3)	0.0328 (4)	0.0004 (3)	0.0180 (4)	0.0023 (3)
O3	0.0399 (4)	0.0302 (4)	0.0372 (4)	−0.0043 (3)	0.0151 (3)	−0.0002 (3)

### Geometric parameters (Å, º)

**Table:**

C1—O1	1.3808 (11)	C17—C18	1.5243 (16)
C1—C6	1.3866 (14)	C17—H17A	0.9900
C1—C2	1.3951 (13)	C17—H17B	0.9900
C2—C3	1.3902 (14)	C18—C19	1.5259 (16)
C2—H2	0.9500	C18—H18A	0.9900
C3—C4	1.3944 (15)	C18—H18B	0.9900
C3—H3	0.9500	C19—C20	1.5205 (15)
C4—C5	1.3898 (14)	C19—H19A	0.9900
C4—C7	1.5024 (14)	C19—H19B	0.9900
C5—C6	1.3923 (14)	C20—C21	1.5272 (15)
C5—H5	0.9500	C20—H20A	0.9900
C6—H6	0.9500	C20—H20B	0.9900
C7—O2	1.4735 (13)	C21—C22	1.5188 (15)
C7—C8	1.5256 (15)	C21—H21A	0.9900
C7—H7	1.0000	C21—H21B	0.9900
C8—C9	1.5043 (14)	C22—C23	1.5244 (17)
C8—H8A	0.9900	C22—H22A	0.9900
C8—H8B	0.9900	C22—H22B	0.9900
C9—N1	1.2854 (13)	C23—H23A	0.9800
C9—C10	1.4616 (13)	C23—H23B	0.9800
C10—C11	1.3991 (13)	C23—H23C	0.9800
C10—C15	1.4005 (13)	C24—O1	1.4744 (11)
C11—C12	1.3825 (14)	C24—C27	1.5160 (14)
C11—H11	0.9500	C24—C25	1.5188 (14)
C12—C13	1.3976 (15)	C24—C26	1.5190 (14)
C12—H12	0.9500	C25—H25A	0.9800
C13—O3	1.3618 (12)	C25—H25B	0.9800
C13—C14	1.3945 (15)	C25—H25C	0.9800
C14—C15	1.3830 (14)	C26—H26A	0.9800
C14—H14	0.9500	C26—H26B	0.9800
C15—H15	0.9500	C26—H26C	0.9800
C16—O3	1.4345 (13)	C27—H27A	0.9800
C16—C17	1.5099 (15)	C27—H27B	0.9800
C16—H16A	0.9900	C27—H27C	0.9800
C16—H16B	0.9900	N1—O2	1.4036 (12)
			
O1—C1—C6	119.95 (9)	C17—C18—H18A	109.2
O1—C1—C2	120.37 (9)	C19—C18—H18A	109.2
C6—C1—C2	119.56 (9)	C17—C18—H18B	109.2
C3—C2—C1	119.74 (9)	C19—C18—H18B	109.2
C3—C2—H2	120.1	H18A—C18—H18B	107.9
C1—C2—H2	120.1	C20—C19—C18	114.01 (10)
C2—C3—C4	121.02 (9)	C20—C19—H19A	108.7
C2—C3—H3	119.5	C18—C19—H19A	108.8
C4—C3—H3	119.5	C20—C19—H19B	108.8
C5—C4—C3	118.59 (9)	C18—C19—H19B	108.7
C5—C4—C7	119.33 (9)	H19A—C19—H19B	107.6
C3—C4—C7	121.87 (9)	C19—C20—C21	113.48 (9)
C4—C5—C6	120.76 (10)	C19—C20—H20A	108.9
C4—C5—H5	119.6	C21—C20—H20A	108.9
C6—C5—H5	119.6	C19—C20—H20B	108.9
C1—C6—C5	120.21 (9)	C21—C20—H20B	108.9
C1—C6—H6	119.9	H20A—C20—H20B	107.7
C5—C6—H6	119.9	C22—C21—C20	112.75 (9)
O2—C7—C4	106.70 (8)	C22—C21—H21A	109.0
O2—C7—C8	104.03 (8)	C20—C21—H21A	109.0
C4—C7—C8	119.41 (10)	C22—C21—H21B	109.0
O2—C7—H7	108.7	C20—C21—H21B	109.0
C4—C7—H7	108.7	H21A—C21—H21B	107.8
C8—C7—H7	108.7	C21—C22—C23	113.74 (10)
C9—C8—C7	101.00 (8)	C21—C22—H22A	108.8
C9—C8—H8A	111.6	C23—C22—H22A	108.8
C7—C8—H8A	111.6	C21—C22—H22B	108.8
C9—C8—H8B	111.6	C23—C22—H22B	108.8
C7—C8—H8B	111.6	H22A—C22—H22B	107.7
H8A—C8—H8B	109.4	C22—C23—H23A	109.5
N1—C9—C10	120.82 (9)	C22—C23—H23B	109.5
N1—C9—C8	113.57 (9)	H23A—C23—H23B	109.5
C10—C9—C8	125.54 (8)	C22—C23—H23C	109.5
C11—C10—C15	118.12 (9)	H23A—C23—H23C	109.5
C11—C10—C9	120.59 (9)	H23B—C23—H23C	109.5
C15—C10—C9	121.29 (9)	O1—C24—C27	103.52 (7)
C12—C11—C10	121.74 (9)	O1—C24—C25	110.07 (8)
C12—C11—H11	119.1	C27—C24—C25	111.21 (9)
C10—C11—H11	119.1	O1—C24—C26	110.96 (8)
C11—C12—C13	119.47 (9)	C27—C24—C26	110.80 (9)
C11—C12—H12	120.3	C25—C24—C26	110.12 (9)
C13—C12—H12	120.3	C24—C25—H25A	109.5
O3—C13—C14	115.85 (9)	C24—C25—H25B	109.5
O3—C13—C12	124.70 (9)	H25A—C25—H25B	109.5
C14—C13—C12	119.45 (9)	C24—C25—H25C	109.5
C15—C14—C13	120.66 (9)	H25A—C25—H25C	109.5
C15—C14—H14	119.7	H25B—C25—H25C	109.5
C13—C14—H14	119.7	C24—C26—H26A	109.5
C14—C15—C10	120.55 (9)	C24—C26—H26B	109.5
C14—C15—H15	119.7	H26A—C26—H26B	109.5
C10—C15—H15	119.7	C24—C26—H26C	109.5
O3—C16—C17	107.12 (9)	H26A—C26—H26C	109.5
O3—C16—H16A	110.3	H26B—C26—H26C	109.5
C17—C16—H16A	110.3	C24—C27—H27A	109.5
O3—C16—H16B	110.3	C24—C27—H27B	109.5
C17—C16—H16B	110.3	H27A—C27—H27B	109.5
H16A—C16—H16B	108.5	C24—C27—H27C	109.5
C16—C17—C18	112.46 (10)	H27A—C27—H27C	109.5
C16—C17—H17A	109.1	H27B—C27—H27C	109.5
C18—C17—H17A	109.1	C9—N1—O2	109.83 (8)
C16—C17—H17B	109.1	C1—O1—C24	117.13 (7)
C18—C17—H17B	109.1	N1—O2—C7	109.13 (7)
H17A—C17—H17B	107.8	C13—O3—C16	118.29 (9)
C17—C18—C19	111.98 (10)		
			
O1—C1—C2—C3	179.22 (9)	C11—C12—C13—C14	−0.15 (17)
C6—C1—C2—C3	3.20 (15)	O3—C13—C14—C15	179.57 (10)
C1—C2—C3—C4	−0.24 (16)	C12—C13—C14—C15	−0.68 (17)
C2—C3—C4—C5	−2.29 (15)	C13—C14—C15—C10	0.94 (17)
C2—C3—C4—C7	172.41 (10)	C11—C10—C15—C14	−0.37 (16)
C3—C4—C5—C6	1.90 (15)	C9—C10—C15—C14	179.85 (10)
C7—C4—C5—C6	−172.94 (10)	O3—C16—C17—C18	177.42 (9)
O1—C1—C6—C5	−179.63 (9)	C16—C17—C18—C19	179.84 (10)
C2—C1—C6—C5	−3.60 (15)	C17—C18—C19—C20	176.39 (10)
C4—C5—C6—C1	1.04 (16)	C18—C19—C20—C21	−178.64 (10)
C5—C4—C7—O2	98.97 (11)	C19—C20—C21—C22	−175.20 (10)
C3—C4—C7—O2	−75.69 (12)	C20—C21—C22—C23	179.67 (11)
C5—C4—C7—C8	−143.67 (10)	C10—C9—N1—O2	−178.73 (9)
C3—C4—C7—C8	41.67 (14)	C8—C9—N1—O2	−1.68 (13)
O2—C7—C8—C9	−14.56 (11)	C6—C1—O1—C24	−91.52 (11)
C4—C7—C8—C9	−133.30 (10)	C2—C1—O1—C24	92.48 (11)
C7—C8—C9—N1	10.69 (13)	C27—C24—O1—C1	176.34 (8)
C7—C8—C9—C10	−172.42 (9)	C25—C24—O1—C1	−64.71 (11)
N1—C9—C10—C11	−1.52 (15)	C26—C24—O1—C1	57.44 (11)
C8—C9—C10—C11	−178.19 (10)	C9—N1—O2—C7	−8.77 (12)
N1—C9—C10—C15	178.25 (10)	C4—C7—O2—N1	142.06 (9)
C8—C9—C10—C15	1.58 (16)	C8—C7—O2—N1	14.95 (12)
C15—C10—C11—C12	−0.47 (16)	C14—C13—O3—C16	−179.53 (10)
C9—C10—C11—C12	179.31 (10)	C12—C13—O3—C16	0.74 (17)
C10—C11—C12—C13	0.72 (17)	C17—C16—O3—C13	179.46 (9)
C11—C12—C13—O3	179.58 (11)		

### Hydrogen-bond geometry (Å, º)

**Table:**

*D*—H···*A*	*D*—H	H···*A*	*D*···*A*	*D*—H···*A*
C26—H26*B*···O2^i^	0.98	2.56	3.4652 (14)	154
C15—H15···O1^ii^	0.95	2.61	3.5542 (12)	173

Symmetry codes: (i) −*x*+3, −*y*+2, −*z*; (ii) −*x*+3, −*y*+1, −*z*.

## References

[bb1] Bezborodov, V., Kauhanka, N. & Lapanik, V. (2004). *Mol. Cryst. Liq. Cryst.* **411**, 1145–1152.

[bb2] Bruker (2012). *APEX2*, *SAINT* and *SADABS*. Bruker AXS Inc., Madison, Wisconsin, USA.

[bb3] Choe, H., Pham, T. T., Lee, J. Y., Latif, M., Park, H., Kang, Y. K. & Lee, J. (2016). *J. Org. Chem.* **81**, 2612–2617. 10.1021/acs.joc.5b02760 26894643

[bb4] El-Khatatneh, N., Vinayaka, A. C., Chandra, , Sadashiva, M. P., Jeyaseelan, S. & Mahendra, M. (2017). *IUCrData*, **2**, x170278.

[bb5] Fader, L. D. & Carreira, E. M. (2004). *Org. Lett.* **6**, 2485–2488. 10.1021/ol0490633 15228310

[bb6] Fritsch, L. & Merlo, A. A. (2016). *ChemistrySelect*, **1**, 23–30.

[bb7] Groom, C. R., Bruno, I. J., Lightfoot, M. P. & Ward, S. C. (2016). *Acta Cryst.* B**72**, 171–179. 10.1107/S2052520616003954 PMC4822653 27048719

[bb8] Hanka, L. J., Gerpheide, S. A., Spieles, P. R., Martin, D. G., Belter, P. A., Coleman, T. A. & Meyer, H. F. (1975). *Antimicrob. Agents Chemother.* **7**, 807–810. 10.1128/aac.7.6.807 PMC429230 1155921

[bb9] Huang, H., Li, F., Xu, Z., Cai, J., Ji, X. & Deng, G.-J. (2017). *Adv. Synth. Catal.* **359**, 3102–3107.

[bb10] Huisgen, R. (1976). *J. Org. Chem.* **41**, 403–419.

[bb11] Kleinbeck, F. & Carreira, E. M. (2009). *Angew. Chem. Int. Ed.* **48**, 578–581. 10.1002/anie.200804645 19067440

[bb12] Lobo, M. M., Viau, C. M., dos Santos, J. M., Bonacorso, H. G., Martins, M. A. P., Amaral, S. S., Saffi, J. & Zanatta, N. (2015). *Eur. J. Med. Chem.* **101**, 836–842. 10.1016/j.ejmech.2015.06.040 26275603

[bb13] Lopes, L. D., Bortoluzzi, A. J., Prampolini, G., dos Santos, F. P., Livotto, P. R. & Merlo, A. A. (2018). *J. Fluor. Chem.* **211**, 24–36.

[bb14] Macrae, C. F., Bruno, I. J., Chisholm, J. A., Edgington, P. R., McCabe, P., Pidcock, E., Rodriguez-Monge, L., Taylor, R., van de Streek, J. & Wood, P. A. (2008). *J. Appl. Cryst.* **41**, 466–470.

[bb15] Martin, D. G., Chidester, C. G., Mizsak, S. A., Duchamp, D. J., Baczynskyj, L., Krueger, W. C., Wnuk, R. J. & Meulman, P. A. (1975). *J. Antibiot.* **28**, 91–93.

[bb16] Muri, D. & Carreira, E. M. (2009). *J. Org. Chem.* **74**, 8695–8712. 10.1021/jo901817b 19839571

[bb17] Orth, R., Böttcher, T. & Sieber, S. A. (2010). *Chem. Commun.* **46**, 8475–8477. 10.1039/c0cc02825h 20936198

[bb18] Ozoe, Y., Asahi, M., Ozoe, F., Nakahira, K. & Mita, T. (2010). *Biochem. Biophys. Res. Commun.* **391**, 744–749. 10.1016/j.bbrc.2009.11.131 19944072

[bb19] Passo, J. A., Vilela, G. D., Schneider, P. H., Ritter, O. M. S. & Merlo, A. A. (2008). *Liq. Cryst.* **35**, 833–840.

[bb20] Pirrung, M. C., Tumey, L. N., Raetz, C. R. H., Jackman, J. E., Snehalatha, K., McClerren, M. L., Fierke, C. A., Gantt, S. L. & Rusche, K. M. (2002). *J. Med. Chem.* **45**, 4359–4370. 10.1021/jm020183v 12213077

[bb21] Samshuddin, S., Butcher, R. J., Akkurt, M., Narayana, B. & Yathirajan, H. S. (2011). *Acta Cryst.* E**67**, o1975–o1976. 10.1107/S1600536811026833 PMC3213434 22091013

[bb22] Sheldrick, G. M. (2008). *Acta Cryst.* A**64**, 112–122. 10.1107/S0108767307043930 18156677

[bb23] Sheldrick, G. M. (2015). *Acta Cryst.* C**71**, 3–8.

[bb24] Spek, A. L. (2009). *Acta Cryst.* D**65**, 148–155. 10.1107/S090744490804362X PMC2631630 19171970

[bb25] Stosic-Grujicic, S., Cvetkovic, I., Mangano, K., Fresta, M., Maksimovic-Ivanic, D., Harhaji, L., Popadic, D., Momcilovic, M., Miljkovic, D., Kim, J., Abed, Y. A. & Nicoletti, F. (2007). *J. Pharmacol. Exp. Ther.* **320**, 1038–1049. 10.1124/jpet.106.109272 17148780

[bb26] Tangallapally, R. P., Sun, D., Rakesh, , Budha, N., Lee, R. E., Lenaerts, A. J., Meibohm, B. & Lee, R. E. (2007). *Bioorg. & Med. Chem. Lett.* **17**, 6638–6642. 10.1016/j.bmcl.2007.09.048 PMC2140235 17937983

[bb27] Tavares, A., Ritter, O. M. S., Vasconcelos, U. B., Arruda, B. C., Schrader, A., Schneider, P. H. & Merlo, A. A. (2010). *Liq. Cryst.* **37**, 159–169.

[bb28] Tavares, A., Schneider, P. H. & Merlo, A. A. (2009). *Eur. J. Org. Chem.* pp. 889–897.

[bb29] Tavares, A., Toldo, J. M., Vilela, G. D., Gonçalves, P. F. B., Bechtold, I. H., Kitney, S. P., Kelly, S. M. & Merlo, A. A. (2016). *New J. Chem.* **40**, 393–401.

[bb30] Westrip, S. P. (2010). *J. Appl. Cryst.* **43**, 920–925.

